# Metabolic interactions enable aerobic degradation of the environmental pollutant BDE-47

**DOI:** 10.1093/ismejo/wrag163

**Published:** 2026-06-27

**Authors:** Piaopiao Pan, Ning-Yi Zhou

**Affiliations:** State Key Laboratory of Microbial Metabolism, Joint International Research Laboratory of Metabolic and Developmental Sciences, and School of Life Sciences and Biotechnology, Shanghai Jiao Tong University, Shanghai 200240, China; State Key Laboratory of Microbial Metabolism, Joint International Research Laboratory of Metabolic and Developmental Sciences, and School of Life Sciences and Biotechnology, Shanghai Jiao Tong University, Shanghai 200240, China

**Keywords:** bioremediation, catabolic pathway, ecological distribution, polybrominated diphenyl ethers, synergistic degradation, synthetic consortium

## Abstract

As a prevalent congener of polybrominated diphenyl ethers (PBDEs), 2,2′,4,4′-tetrabromodiphenyl ether (BDE-47) poses significant environmental and health risks due to its persistence and bioaccumulation. However, the limited understanding of the microbial degradation mechanism of BDE-47 has hindered the development of effective bioremediation strategies. Here, we decipher an aerobic catabolic pathway of BDE-47 mediated by metabolic relay within a synthetic consortium composed of two environmental isolates, *Rhizorhabdus wittichii* YL-JM2C and *Cupriavidus necator* JMP134. Bioaugmentation with this consortium achieved complete removal of BDE-47 in real wastewater samples. The molecular basis underlying this cooperative degradation was elucidated through the heterologous expression and functional characterization of key enzymes involved. Namely, the dioxygenase TcsAaAb from strain YL-JM2C catalyzed the initial conversion of BDE-47 into 2,4-dibromophenol (2,4-DBP) and 3,5-dibromocatechol (3,5-DBC). As a dead-end intermediate in strain YL-JM2C, the former (2,4-DBP) was subsequently transformed into the latter (3,5-DBC) by the hydroxylase TfdB from strain JMP134. The resulting 3,5-DBC was catabolized through the downstream *ortho*-cleavage pathway present in both strains. These key enzymes for BDE-47 degradation coexist across diverse environments, including soil, seawater, and marine sediments. Global marine metagenomic profiling revealed a significant enrichment of these catabolic signatures in the Mariana Trench, implying that microorganisms in the hadal zone possess the genetic potential for PBDE catabolism. This study unveils previously unrecognized aerobic catabolic mechanisms for BDE-47 within natural ecosystems, offering promising bioremediation strategies for PBDE-contaminated environments.

## Introduction

Polybrominated diphenyl ethers (PBDEs) are a class of brominated flame retardants extensively incorporated into consumer products including electronic equipment, textiles, plastics, and building materials [1, 2]. As additives rather than covalently bound components, PBDEs can be inevitably released into the environment over time through abrasion, aging, or heat exposure [3]. Consequently, PBDE congeners have been widely detected in environmental matrices and biological tissues over recent decades [4–7]. Due to their environmental persistence, bioaccumulative properties, and potential toxicity, PBDEs have been listed under the Stockholm Convention on Persistent Organic Pollutants (POPs) [8], reflecting global concern for their ecological and human health impacts. Among 209 congeners of PBDEs, 2,2′,4,4′-tetrabromodiphenyl ether (BDE-47) is one of the most prevalent and concerning congeners [9]. It is not only a primary component of commercial Penta-BDE mixtures but also a major debromination product of higher-brominated congeners [10]. Previous studies have demonstrated that chronic exposure to BDE-47 can induce a variety of adverse health effects, including hepatotoxicity, neurotoxicity, and reproductive toxicity [11–13]. Therefore, it is essential to develop effective strategies for eliminating BDE-47 from contaminated environments to mitigate its ecological and health risks.

Generally, PBDEs are primarily eliminated through photodegradation, chemical redox, and microbial degradation [14–16]. Although the former two are efficient, they often require complex conditions and risk producing toxic by-products [17, 18]; thus, microbial degradation is increasingly favored as a more eco-friendly and cost-effective strategy [19]. Microbial degradation of PBDEs has previously been investigated under both anaerobic and aerobic conditions [20–22]. The anaerobic degradation of PBDEs predominantly occurs via reductive debromination, wherein highly brominated diphenyl ethers are gradually converted into lower-brominated congeners by dehalogenases [23, 24]. Nevertheless, this process is typically slow and rarely achieves complete mineralization of PBDEs. In contrast, aerobic degradation is generally faster and can facilitate more extensive catabolism of PBDEs [25]. Thus far, several aerobic strains have been found to be capable of degrading BDE-47, including *Pseudomonas stutzeri* [26], *Pseudomonas aeruginosa* [27], *Stenotrophomonas* sp. [28], and *Acinetobacter pittii* [29]. However, previous studies largely focused on the isolation of degrading strains, characterization of degradation capacity, and identification of metabolic products. Although several intermediates derived from BDE-47 degradation have been identified [25, 27], such as debrominated metabolites, hydroxylated metabolites, and brominated phenols, the complete degradation pathway of BDE-47 remains poorly understood. Moreover, the genes and enzymes responsible for BDE-47 degradation have yet to be elucidated, hindering a comprehensive understanding of its catabolic mechanisms.

Microbial synthetic communities (SynComs) are artificially designed microbial consortia composed of strains representing the taxonomic and functional characteristics of natural microbiomes, allowing controlled investigation of microbial interactions and ecosystem functions [30, 31]. SynComs can be designed based on two main strategies: top-down and bottom-up approaches [32]. The top-down approach starts from complex natural microbial communities and simplifies them through retaining key functional members, whereas the bottom-up approach assembles SynComs from well-characterized individual strains selected based on their complementary metabolic functions or ecological traits [33, 34]. In recent years, SynComs have been successfully employed for diverse objectives, including soil fertility enhancement [35], plant disease suppression [36], plant growth promotion [37], and environmental pollutant remediation [38]. For example, in a two-member SynCom composed of strains X-1 and 7D-2, X-1 metabolizes bromoxynil octanoate to release bromoxynil for 7D-2, whereas 7D-2 secretes xanthine and mannose that support X-1 growth [39]. Aerobic degradation of BDE-47 has primarily been studied using single bacterial strains, generally accompanied by the accumulation of toxic intermediates including hydroxylated diphenyl ethers (such as 5-OH-BDE-47 and 6-OH-BDE-47), which exhibit even higher toxicity than their parent compound [27]. Compared with single-strain systems, SynComs can improve degradation performance via multiple cooperative mechanisms with new traits, such as synergistic metabolism, cross-feeding, and detoxification of intermediates [40–42]. Therefore, constructing functional SynComs may represent a promising strategy for the extensive elimination of BDE-47 from contaminated environments.

In this study, we achieved the complete degradation of BDE-47 through metabolic synergy within a synthetic consortium engineered via a bottom-up strategy. Biodegradation assays and biochemical characterization demonstrated that TcsAaAb-initiated catabolism of BDE-47 by *Rhizorhabdus wittichii* YL-JM2C leads to the accumulation of the dead-end intermediate 2,4-dibromophenol (2,4-DBP), which is eliminated by incorporating the hydroxylase TfdB from *Cupriavidus necator* JMP134. Bioaugmentation with this consortium facilitated the efficient removal of BDE-47 within complex wastewater matrices. Furthermore, bioinformatic analyses revealed the co-occurrence of the enzymes involved in BDE-47 degradation across diverse natural niches, especially within marine ecosystems. These findings advance our understanding of BDE-47 aerobic catabolism and the microbially mediated cycling of anthropogenic PBDEs in the global ocean.

## Materials and methods

### Chemicals, bacterial strains, and plasmids

All chemicals and culture media were purchased from Aladdin (Shanghai, China) with the following exceptions: 3,5-dibromocatechol (3,5-DBC; Bide Pharmatech, China), nicotinamide adenine dinucleotide phosphate (NADPH; Beyotime, China), R2A medium (Hope Bio-Technology, China), and Chromatography-grade solvents (InnoChem, China). Bacterial strains and plasmids used in this study are summarized in Table S1. *Escherichia coli* strains were cultured in lysogeny broth (LB) at 37°C, supplemented with kanamycin (50 μg/mL) when necessary.

### Growth and degradation experiments

*R. wittichii* YL-JM2C [43] was pre-grown to exponential phase in R2A, washed with 50 mM Tris–HCl buffer (pH 7.4), and inoculated (1%, v/v) into minimal salts medium (MSM, pH 7.0) containing BDE-47 (18 μM) and yeast extract (0.04%, w/v) [44]. All assays were performed in triplicate at 30°C with shaking at 180 rpm, with uninoculated medium as an abiotic control. Bacterial growth (OD_600_) and BDE-47 dissipation were monitored via a microplate reader and high-performance liquid chromatography (HPLC), respectively.

For intermediate identification, whole-cell biotransformation was performed. Strain YL-JM2C was cultivated in R2A supplemented with 10 μM triclosan (TCS) or glucose to exponential phase. The induced cells were then harvested, washed, and resuspended in MSM (OD_600_ = 4.0) containing 50 μM BDE-47. The assay was conducted at 30°C with shaking at 180 rpm, with sampling at defined intervals to monitor substrate depletion and product formation.

### Gene cloning, protein expression, and purification

Target genes were amplified using primers listed in Table S2 and cloned into pET-28a(+) using the ClonExpress MultiS One Step cloning kit (Vazyme, China). The resulting recombinant products were transformed into *E. coli* DH5α competent cells via heat shock. After verification by sequencing, correct plasmids were introduced into *E. coli* BL21(DE3) for protein expression. The *E. coli* cells were grown at 37°C in LB containing 50 μg/mL kanamycin to an OD_600_ of 0.6, then induced with 0.3 mM Isopropyl β-D-thiogalactopyranoside (IPTG) at 16°C for 14 h. Harvested cells were resuspended in 50 mM Tris–HCl (pH 7.4), lysed via sonication, and clarified by centrifugation (15 000 × *g*, 45 min, 4°C). His-tagged proteins were purified using a 5 mL HisTrap HP column (GE Healthcare) and eluted with 250 mM imidazole. Protein concentration and purity were determined via Nano-300 spectrophotometer (Allsheng, China) and SDS-PAGE, respectively.

### Functional characterization of BDE-47 degradation enzymes

The TcsAaAb activity in BDE-47 transformation was investigated using a whole-cell biotransformation assay. *E. coli* BL21(DE3) harboring pET-*tcsAaAb* was cultivated and induced as mentioned above. The cells were harvested by centrifugation and resuspended in MSM to an OD_600_ of 4.0. Subsequently, 50 μM BDE-47 was added to the cell suspension and the mixture was incubated at 30°C with shaking for 6 h.

The catalytic activities of TfdB homologs were determined by *in vitro* enzyme assays. Enzyme assays of TfdB homologs were conducted in Tris–HCl buffer (pH 7.4) containing 15 μg purified TfdB, 200 μM NADPH, and 10 μM flavin adenine dinucleotide (FAD), and the reaction was initiated by adding 200 μM substrate. The activity of TfdB homologs toward all substrates was determined by monitoring the decrease in absorbance at 340 nm using a Lambda 25 spectrophotometer (PerkinElmer/Cetus, Norwalk, CT). The molar extinction coefficient of NADPH at 340 nm is 6220 M^−1^ cm^−1^ [45]. One unit of enzyme activity (U) is defined as the amount of enzyme catalyzing the formation of 1 μM product per minute, and the specific activity was expressed in units per milligram of protein. The activities of TcsC, TcsD, TcsE, TcsF, PcaIJ, and PcaF in the sequential catabolism of 3,5-DBC were individually verified according to described methods [46–48].

### Degradation of BDE-47 by a bacterial consortium

*R. wittichii* YL-JM2C and *C. necator* JMP134 were grown separately in R2A to exponential phase, harvested, and resuspended in MSM (pH 7.0) to an OD_600_ of 4.0. A synthetic consortium was established by mixing equal volumes of both cell suspensions, followed by the addition of BDE-47. The mixtures were incubated at 30°C with shaking at 180 rpm in triplicate, with samples collected periodically for HPLC analysis. To optimize BDE-47 degradation by the synthetic consortium, parameters including temperature (16–50°C) and pH (5.0–10.0) were evaluated. Assays were conducted by varying the target parameter, with all other conditions kept constant (30°C, pH 7.0, and 180 rpm).

The bioremediation potential of the synthetic consortium against BDE-47 was evaluated in real wastewater. Wastewater samples were collected from wastewater treatment plants (WWTPs) across three different cities (Table S3). For each sample, a 5-mL wastewater microcosm was established and amended with 20 μM BDE-47 as the target pollutant for subsequent bioremediation. Both strains were prepared as described above (OD_600_ = 1.0) and inoculated (5%, v/v) into the microcosms. Yeast extract (0.04%, w/v) was added as an auxiliary carbon source. Non-inoculated microcosms served as controls. Residual BDE-47 concentrations were quantified via HPLC at 0, 2, and 4 days.

### Quantification of bacterial abundance during BDE-47 degradation

Cell pellets from samples collected at different time points were harvested by centrifugation (11 500 × *g*, 2 min, 15°C). Total genomic DNA was extracted using the TIANamp Bacteria DNA Kit according to the manufacturer’s instructions (TIANGEN, Beijing, China). Strain-specific primers targeting the 16S rRNA gene regions of strain YL-JM2C and strain JMP134 (Table S2) were used in quantitative PCR (qPCR) assays, which were performed on a CFX96 Real-Time PCR Detection System (Bio-Rad). For absolute quantification, standard curves were generated using serial dilutions of plasmid DNA containing the targeted 16S rRNA gene fragments of each strain (ranging from 10^4^ to 10^9^ copies/mL). The abundance of each strain was calculated based on the Ct values using the corresponding standard curve.

### Bioinformatic analysis of key enzymes for BDE-47 degradation

TcsAa homologs were retrieved from the NCBI NR protein database using DIAMOND [49], applying a threshold of 35% identity and 70% sequence coverage. Genomes of strains harboring tcsAa were subsequently downloaded to examine the presence of TcsAb, TfdB, and TcsC. Similarly, TfdB homologs were identified using a stricter threshold (50% identity and 70% coverage). A phylogenetic tree was then constructed using these TfdB homologs, along with reference sequences TfdBI, TfdBII, and RwTfdB (Table S4). Proteins clustering with TfdBI and TfdBII but forming a distinct branch from RwTfdB were inferred to be potential TfdB candidates. The taxonomic and environmental origins of these enzymes were manually retrieved from NCBI database. The phylogenetic tree was constructed using IQ-TREE2 [50] and visualized with ChiPlot (https://www.chiplot.online/).

To explore the global distribution of the key functional genes involved in BDE-47 degradation, a total of 1410 marine metagenomes across the global ocean were analyzed based on previously assembled datasets [51]. Homologs of TcsAaAb, TfdB, and TcsC were identified using the same criteria and thresholds as described above. The relative abundances of genes encoding these target enzymes were calculated and expressed as Reads Per Kilobase per Million mapped reads (RPKM) [51].

### Genome sequencing and analysis of strain YL-JM2C

Genomic DNA of strain YL-JM2C was extracted and sequenced using a hybrid Nanopore and Illumina approach (Magigene, China). The genome was assembled using Unicycler [52], followed by polishing with Arrow [53]. Gene prediction and annotation were carried out using Prokka [54] for protein-coding genes, tRNAscan-SE (v1.4) [55] for tRNA genes, and RNAmmer (v1.2) [56] for rRNA genes. Average nucleotide identity (ANI) between strain YL-JM2C and representative strains was calculated using JSpeciesWS via the ANIb algorithm [57], with results visualized as a heatmap using ChiPlot.

### Microbial diversity analysis of wastewater samples

Microbial diversity sequencing and analysis were performed by Tsingke Biotechnology Co., Ltd. (Beijing, China). For each treatment, triplicate samples from each time point were pooled and centrifuged for biomass collection. Total genomic DNA was extracted using the TGuide S96 Magnetic DNA Kit (Tiangen, China). The V3-V4 region of the 16S rRNA gene was amplified using primers 341F/806R and sequenced on a NovaSeq 6000 System (Illumina). Raw reads were quality-filtered and trimmed via Trimmomatic (v0.33) [58] and Cutadapt (v1.8.3) [59]. The DADA2 plugin in QIIME2 (v2020.6) was employed for denoising and generating amplicon sequence variants (ASVs) [60]. Taxonomic assignment was performed with a naive Bayes classifier trained on the SILVA database (v138) [60]. Taxonomic profiles were visualized using ggplot2 package in R.

### Analytical methods

BDE-47 and 2,4-DBP were quantified using an Agilent Infinity II HPLC system at detection wavelengths of 226 nm and 280 nm, respectively. The column temperature was maintained at 35°C, and the injection volume was 10 μL. The detailed chromatographic conditions are provided in Table S5. Prior to analysis, each sample was mixed with an equal volume of acetonitrile, centrifuged, and filtered through a 0.22-μm nylon membrane. Bromide ion quantification was performed on an ICS-5000+/900 ion chromatography system (Thermo Fisher Scientific, USA).

Transformation products were identified using liquid chromatography–mass spectrometry (LC–MS) and gas chromatography–mass spectrometry (GC–MS). LC–MS analyses were performed on an Agilent 1290 Infinity II system coupled with a time-of-flight mass spectrometer, with chromatographic conditions as described in Table S5. MS data were acquired in negative ion mode (*m/z* 50–800). GC–MS analyses were conducted using an Agilent 7890B gas chromatograph coupled with an Agilent 5977B mass spectrometer. The oven temperature was programmed as follows: an initial temperature of 70°C was maintained for 2 min, then increased to 130°C at 5°C/min, followed by a ramp to 180°C at 10°C/min. The temperature was further elevated to 285°C at 40°C/min and held for 5 min. Samples were extracted with isooctane as described previously [21], and the collected fractions were derivatized with an equal volume of *N,O*-bis(trimethylsilyl)trifluoroacetamide at 60°C for 30 min before GC–MS analysis.

## Results and discussion

### Degradation of BDE-47 by triclosan-degrading bacterium YL-JM2C

**Figure 1:**
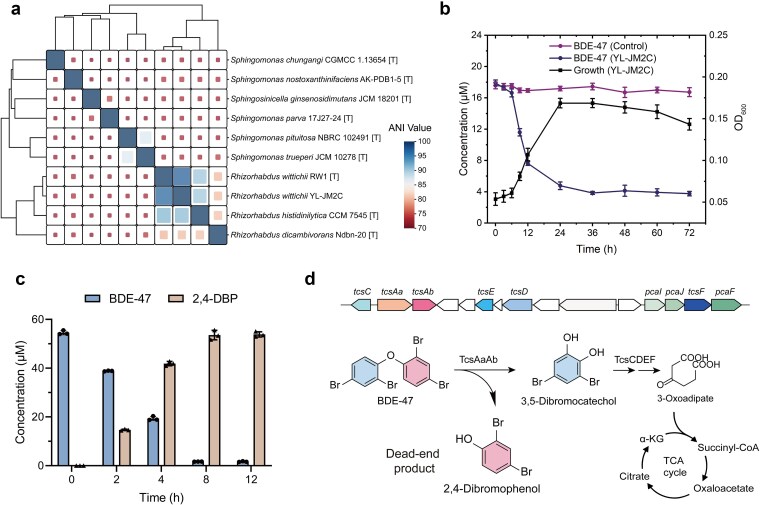
Phylogenetic analysis of *R. wittichii* YL-JM2C and its degradation of BDE-47. (a) The ANI values between strain YL-JM2C and other nine strains. Type strains are marked with “[T]”. (b) Growth of strain YL-JM2C and the dissipation of BDE-47. (c) Whole-cell biotransformation of BDE-47 by strain YL-JM2C. The concentrations of BDE-47 and 2,4-DBP were measured by HPLC. (d) Proposed pathway of BDE-47 catabolism in strain YL-JM2C.

Strain YL-JM2C is a TCS degrader isolated from a wastewater treatment plant, which was initially assigned to the genus *Sphingomonas* based on 16S rRNA gene sequence [43]. However, whole-genome analysis revealed that strain YL-JM2C shared the highest ANI value (∼97%) with *R. wittichii* RW1 (Fig. 1a), whereas the ANI values with several representative members of the genus *Sphingomonas* were all below 80% (Table S6). Hence, strain YL-JM2C was reclassified as *R. wittichii* YL-JM2C. Given the structural similarity between TCS and BDE-47, we hypothesized that strain YL-JM2C might also possess the ability to degrade BDE-47. When strain YL-JM2C was cultivated in yeast extract (0.04%, w/v)-containing MSM supplemented with BDE-47, its biomass concentration (OD_600_) increased from 0.05 to 0.15 within the first 24 h and then slightly declined to 0.14 at the end of cultivation (Fig. 1b). Concurrently, the concentration of BDE-47 decreased from 18 to 4 μM, with the most rapid degradation occurring during the exponential growth phase of this strain. In contrast, the concentration of BDE-47 remained unchanged in the uninoculated control (Fig. 1b).

To identify and quantify the intermediates formed during BDE-47 biodegradation, whole-cell biotransformation assays were conducted. A single new peak was observed by LC–MS analysis in the biotransformation samples, which was identified as 2,4-DBP based on the comparison of retention time (12.0 min) and mass spectrum (*m/z*: 250.8514; [M-H]^−^) with those of authentic standard (Fig. S1). The degradation of BDE-47 by strain YL-JM2C was accompanied by the stoichiometric formation of 2,4-DBP, which was considered a dead-end product (Fig. 1c). The accumulation of this monocyclic compound suggests that cleavage of the ether bond in BDE-47 occurred during the biodegradation process. A previous study has demonstrated that TCS degradation is initiated by the angular dioxygenation of the dichlorinated benzene ring, leading to the spontaneous cleavage of the ether bond into two monocyclic intermediates [61]. Apparently, this transformation pattern of BDE-47 closely resembles the initial degradation step of TCS, implying that strain YL-JM2C may utilize the same catabolic enzymes for the degradation of both compounds. Moreover, the degradation rate of BDE-47 was significantly accelerated when strain YL-JM2C was pretreated with TCS compared with glucose-pretreated cells (Fig. S2), further supporting the involvement of TCS catabolic genes in BDE-47 degradation. Based on these findings, we propose a putative aerobic degradation pathway of BDE-47 in strain YL-JM2C (Fig. 1d).

### Conversion of BDE-47 to 2,4-DBP and 3,5-DBC by Rieske dioxygenase TcsAaAb

TcsAaAb from strain YL-JM2C is a two-component NAD(P)H-dependent Rieske dioxygenase that has been demonstrated to catalyze the angular dioxygenation of TCS [61]. It is likely that the same enzyme catalyzed the initial reaction of BDE-47 degradation mentioned above. To confirm this, we assessed the transformation ability of *E. coli* BL21(DE3) cells harboring the recombinant plasmid pET-*tcsAaAb* toward BDE-47. As expected, the recombinant cells converted BDE-47 into two intermediates, which were identified as 2,4-DBP and 3,5-DBC by GC–MS through comparison with authentic standards (Fig. S3). Although previous studies have frequently detected bromophenols such as 2,4-DBP and 4-bromophenol (4BP) as the major metabolites during BDE-47 transformation [25, 27], 3,5-DBC has never been reported as one of the intermediates. The detection of these two intermediates in this study provides direct evidence for ether bond cleavage in BDE-47 and suggests that TcsAaAb initiates a previously unrecognized pathway for BDE-47 degradation. Although several aerobic bacterial strains have been reported to degrade PBDEs, no enzyme responsible for their aerobic degradation has been functionally characterized. The functional identification of TcsAaAb toward BDE-47 fills this knowledge gap and expands our understanding of the enzymatic process involved in aerobic PBDE degradation. When whole-cell biotransformation of BDE-47 was performed using strain YL-JM2C, only 2,4-DBP was accumulated and no 3,5-DBC was detected, presumably due to its rapid further catabolism by this strain. This observation motivated our investigation of the 3,5-DBC degradation capacity of TcsC, a chlorocatechol 1,2-dioxygenase encoded by a gene adjacent to *tcsAaAb*. TcsC converted 3,5-DBC into the corresponding muconic acid (Fig. S4), which was further catabolized by TcsDEF (Fig. S5) and subsequently channeled into the TCA cycle via the β-ketoadipate pathway catalyzed by PcaIJ and PcaF (Fig. S6). Taken together, these results demonstrate that TcsAaAb is responsible for the initial step of BDE-47 degradation, catalyzing its conversion into 3,5-DBC and 2,4-DBP. The intermediate 3,5-DBC can be further catabolized, whereas 2,4-DBP accumulates as a dead-end metabolic product in strain YL-JM2C.

### Elimination of 2,4-DBP by TfdB from *C. necator* JMP134

Although strain YL-JM2C is capable of degrading BDE-47, a significant concern is the generation of the toxic metabolite 2,4-DBP. Extensive studies have demonstrated that 2,4-DBP exhibits toxic effects on organisms, including induction of oxidative stress and cellular damage, disruption of endocrine function, and impairment of development and reproduction [62]. Hence, the accumulation of 2,4-DBP necessitates the development of effective strategies for its elimination to achieve the thorough remediation of BDE-47 contamination. *C. necator* JMP134 is a well-characterized bacterial strain known for its ability to mineralize the herbicide 2,4-dichlorophenoxyacetic acid (2,4-D) [63]. The genes responsible for 2,4-D catabolism are located on the plasmid pJP4, including *tfdA, tfdBI, tfdCIDIEIFI, tfdBII*, and *tfdDIICIIEIIFII* (Fig. S7) [64]. A key intermediate in the 2,4-D degradation pathway is 2,4-dichlorophenol (2,4-DCP), which shares a high structural similarity with 2,4-DBP, differing only in the halogen substituents (chlorine versus bromine). This structural analogy suggested that TfdBI and TfdBII, the 2,4-DCP hydroxylases from strain JMP134, might also catalyze the transformation of 2,4-DBP. Furthermore, BLAST analysis revealed that strain YL-JM2C harbors a homolog of TfdB with over 50% sequence identity, designated RwTfdB.

**Figure 2:**
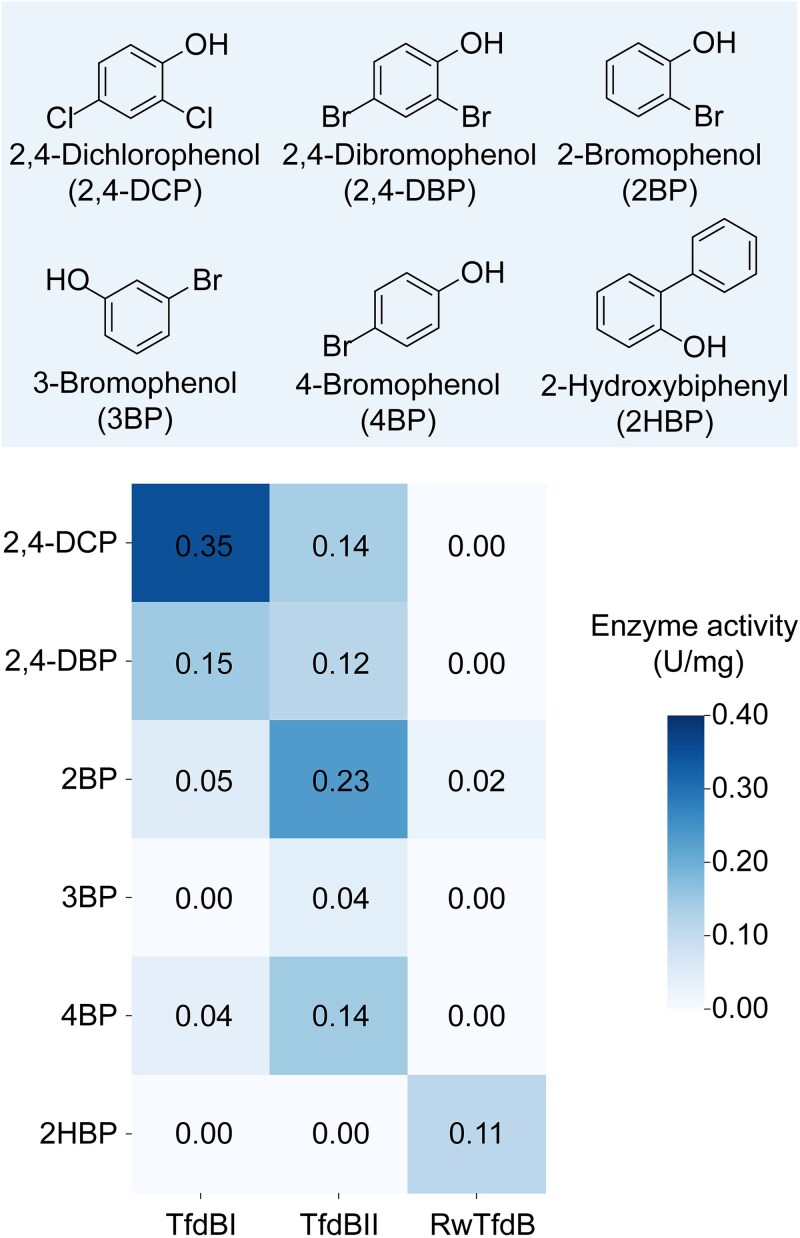
Specific activities of purified TfdB homologs toward different substrates. TfdBI and TfdBII from *C. necator* JMP134 are able to convert 2,4-DBP to 3,5-DBC, but RwTfdB from *R. wittichii* YL-JM2C is not.

To evaluate their possible catalytic capabilities toward 2,4-DBP, whole-cell biotransformation assays were performed using *E. coli* cells expressing TfdBI (BL21-TfdBI), TfdBII (BL21-TfdBII), or RwTfdB (BL21-RwTfdB). The HPLC chromatogram revealed the appearance of a new peak in the reaction mixtures of BL21-TfdBI and BL21-TfdBII, corresponding to the retention time of authentic 3,5-DBC (Fig. S8). In contrast, no additional peaks besides the substrate were detected in the BL21-RwTfdB reaction mixture. To further confirm that RwTfdB was successfully expressed and active, we purified all three TfdB homologs (Fig. S9) and assessed their catalytic activities toward a range of potential substrates. The results demonstrated that RwTfdB was indeed active but towards 2-bromophenol (2BP) and 2-hydroxybiphenyl (2HBP), rather than 2,4-DBP (Fig. 2). This observation further confirms inability of the strain to degrade 2,4-DBP, resulting in its accumulation. In contrast, TfdBI and TfdBII from strain JMP134 were active against all tested substrates except 2HBP. Furthermore, strain JMP134 was able to utilize 2,4-DBP as the sole carbon source for growth (Fig. S10), indicating that TfdB is active *in vivo* and that the downstream catabolic pathway is functional. Therefore, *C. necator* JMP134 represents a promising partner of strain YL-JM2C for eliminating 2,4-DBP produced during BDE-47 degradation.

### Construction of a BDE-47 degrading consortium

**Figure 3:**
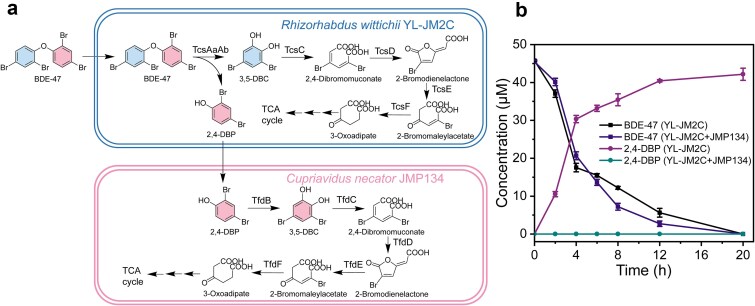
Complete degradation of BDE-47 by a synthetic consortium. (a) Schematic diagram of BDE-47 synergistic degradation by the consortium of *R. wittichii* YL-JM2C and *C. necator* JMP134. (b) Degradation of BDE-47 and the formation of 2,4-DBP by the single strain (YL-JM2C) or the bacterial consortium (YL-JM2C + JMP134), where 2,4-DBP accumulates in the former but complete degradation is achieved in the latter.

To achieve complete degradation of BDE-47 without 2,4-DBP accumulation, a bacterial consortium consisting of *R. wittichii* YL-JM2C and *C. necator* JMP134 was constructed. A catabolic interaction model for the synergistic degradation of BDE-47 was proposed based on gene annotation and functional characterization (Fig. 3a). The Rieske dioxygenase TcsAaAb from strain YL-JM2C initiated the transformation of BDE-47 into 3,5-DBC and 2,4-DBP, the latter being further converted into 3,5-DBC by TfdB from strain JMP134. Because both strains possess downstream gene clusters responsible for 3,5-DBC degradation (*tcsCDEF* in strain YL-JM2C and *tfdCDEF* in strain JMP134), their combined catabolic capacities enabled the complete mineralization of BDE-47. As expected, the consortium successfully eliminated BDE-47 after 20 h without detectable 2,4-DBP (Fig. 3b). In contrast, when strain YL-JM2C was inoculated alone, BDE-47 disappeared within 20 h but accompanying by stoichiometric accumulation of 2,4-DBP. Transwell assays revealed that the synergistic degradation of BDE-47 without 2,4-DBP accumulation persisted even when the two strains were cultured in separate compartments (Fig. S11), demonstrating that this metabolic relay is mediated by cross-feeding of diffusible metabolites rather than direct physical contact. These results highlight the necessity of catabolic relay between strain YL-JM2C and *C. necator* JMP134 in achieving the complete degradation of BDE-47. The cooperative action of TfdB in JMP134 efficiently funneled this toxic intermediate 2,4-DBP into the 3,5-DBC catabolic pathway, thereby preventing its accumulation and ensuring complete degradation.

Multiple rounds of substrate supplementation were performed to further evaluate the functional stability and synergistic interaction of the synthetic consortium, and the abundances of the two strains were monitored by qPCR throughout the process. Within a degradation cycle, the abundance of strain YL-JM2C increased first, followed by an increase in strain JMP134 when BDE-47 was nearly depleted (Fig. S12). This sequential growth pattern is consistent with the proposed metabolic relay. Efficient degradation was maintained throughout all three cycles, and no competitive exclusion or significant biomass decline of either strain was observed. These results indicate that the synthetic consortium supports sustained metabolic relay for BDE-47 degradation.

Temperature and pH are key environmental variables influencing microbial activity, directly determining the efficacy of bioremediation. Investigating their effects helps define the operational range of the consortium and assess its applicability in both natural and engineered systems. The consortium exhibited optimal BDE-47 degradation at 30°C, achieving 93.7% removal within 12 h (Fig. S13). The degradation efficiency of BDE-47 was moderate at 25°C (73.6%) and low at 16°C (19.8%), whereas higher temperatures strongly inhibited degradation, with activity being negligible at 37°C (3.5%) and completely absent at 50°C. In comparison with temperature, pH exerted a relatively minor effect on BDE-47 degradation. High removal efficiencies (>85%) were observed within the pH range of 6–8, whereas degradation rate decreased under strongly alkaline conditions (77.7% at pH 9 and 72% at pH 10) and was lowest (46%) at pH 5. Overall, the consortium performed the optimal BDE-47 degradation at 30°C and pH 7, whereas both lower or higher temperatures and pH values significantly impaired degradation.

### Performance of the synthetic consortium in the real wastewater

**Figure 4:**
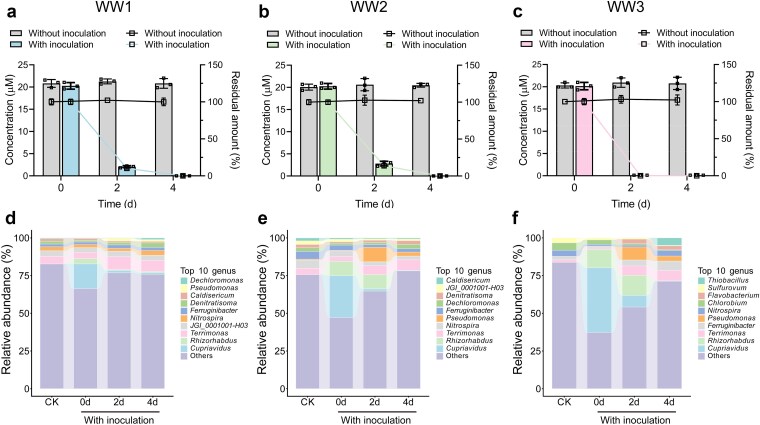
Bioremediation of BDE-47 in wastewater samples through addition of a synthetic consortium. (a–c) Degradation efficiency of BDE-47 by the synthetic consortium in wastewater samples. (d–f) Relative abundance of microbial genera in the wastewater samples, measured at days 0, 2, and 4 during the bioremediation process. CK represents the microbial composition of wastewater samples without bacterial inoculation at day 0.

To verify the remediation capability of the synthetic consortium for BDE-47 in real wastewater, biodegradation experiments were conducted with samples collected from different WWTPs inoculated with the synthetic consortium. The synthetic consortium was inoculated at 5% (v/v) into wastewater samples spiked with 20 μM BDE-47. After 2 days of incubation, the residual concentrations of BDE-47 in WW1 and WW2 were 2.1 and 2.8 μM, respectively, whereas BDE-47 in WW3 was completely removed (Fig. 4a–c). Complete removal of BDE-47 was achieved in all three cultures after 4 days. In contrast, negligible degradation occurred in wastewater without inoculation of the consortium, indicating that the indigenous microorganisms in these WWTP samples lacked the ability to degrade BDE-47, and that its removal was primarily due to the introduced consortium.

To evaluate the viability of the synthetic consortium and the dynamics of indigenous microorganisms in the WWTP samples, we analyzed the microbial community composition at different time points during the remediation process. Before the introduction of the synthetic consortium, the genera *Rhizorhabdus* and *Cupriavidus* were nearly undetectable in all samples. In WW1, the relative abundance of *Rhizorhabdus* was 3.44%, 0.55%, and 0.81% on days 0, 2, and 4, respectively, whereas *Cupriavidus* accounted for 16.47%, 1.53%, and 0.99% on the same days (Fig. 4d). In addition, several indigenous genera exhibited noticeable shifts. The relative abundance of *Terrimonas* increased during BDE-47 degradation compared with the initial community. As a core taxon widely distributed in wastewater treatment systems [65], *Terrimonas* possesses the capacity to degrade complex organic matter [66] and secrete extracellular polymers for cell aggregate formation [67]. Their enrichment during incubation may reflect the utilization of metabolites released during BDE-47 degradation, as well as enhanced cellular protection against toxic stress. The microbial composition on day 4 more closely resembled the pre-introduction state compared to that observed on days 0 or 2. Similar temporal abundance patterns were observed in the other two WWTP samples (Fig. 4e and f). These results indicate that the exogenous consortium largely disappeared after the pollutant BDE-47 was eliminated. The introduced consortium failed to achieve long-term colonization in the wastewater samples, likely due to the depletion of available carbon source and competition with the native microorganisms. To achieve long-term colonization and sustained degradation, immobilization of the bacterial consortium could be a potential strategy if required [68, 69].

### Ecological distribution of key BDE-47 degradation enzymes

**Figure 5:**
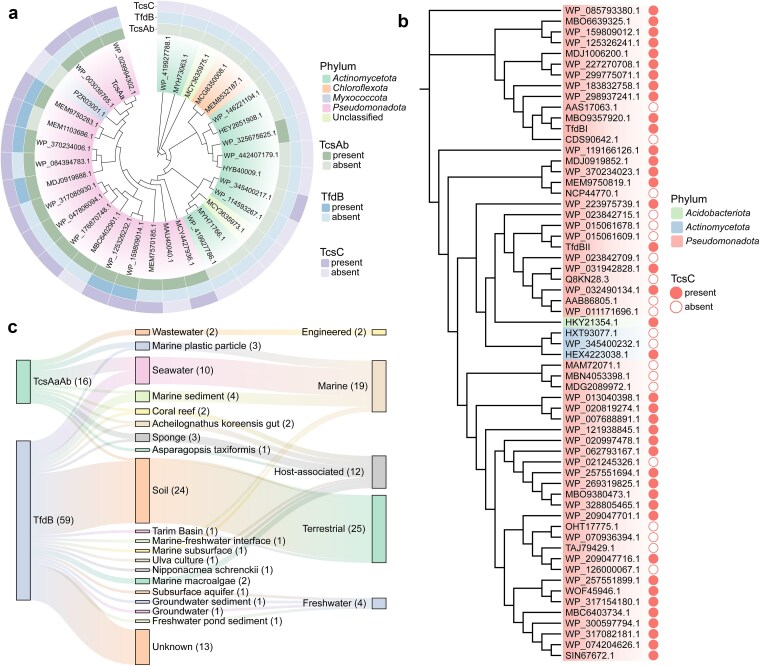
Taxonomic origin and environmental distribution of key enzymes involved in BDE-47 degradation. (a) Phylogenetic tree of TcsAa homologs. The outer ring indicates the presence or absence of TcsAb, TfdB, and TcsC homologs in the bacterial strains harboring TcsAa homologs. (b) Phylogenetic tree of TfdB homologs. The circle symbolizes the presence or absence of TcsC homologs among the TfdB-harboring strains. (c) Sankey diagram shows the environmental distribution of TcsAaAb and TfdB homologs.

As indicated, BDE-47 degradation proceeds through the sequential actions of the dioxygenase TcsAaAb (catalyzing the conversion of BDE-47 into 2,4-DBP and 3,5-DBC) from *R. wittichii* YL-JM2C and the hydroxylase TfdB (catalyzing the conversion of 2,4-DBP into 3,5-DBC) from *C. necator* JMP134, yielding two molecules of 3,5-DBC that subsequently enter downstream catabolism via the dioxygenase TcsC (catalyzing the *ortho*-cleavage of 3,5-DBC). To assess the BDE-47 degradation capacity of microbes across diverse environments, the distribution of TcsAaAb, TfdB, and TcsC homologs was investigated. A total of 32 TcsAa homologs were retrieved from NCBI NR database based on sequence threshold (identity >35% and coverage >70%) (Fig. 5a, Table S7). Conserved domain analysis confirmed that all retrieved TcsAa homologs possess the characteristic Rieske [2Fe-2S] motif and non-heme iron-coordinating residues (Fig. S14A), and phylogenetic analysis showed that they form a monophyletic clade with the reference TcsAa (Fig. S14B), supporting the validity of the 35% identity threshold. Because TcsAb is indispensable for the dioxygenase system that initiates BDE-47 degradation, bacterial genomes harboring TcsAa homologs were downloaded to determine the presence of TcsAb homologs. Of the 29 TcsAa-containing strains, only 16 also harbored TcsAb homologs, 15 of which belonged to *Pseudomonadota* and one to *Actinomycetota*. Intriguingly, five strains possessed the complete set of TcsAaAb, TfdB, and TcsC, implicating their potential for complete degradation of BDE-47 independently. In addition, TfdB candidates were identified through a combination of BLASTP and phylogenetic analysis, and their microbial lineages were investigated as well (Table S8, Fig. S15). *Pseudomonadota* constituted the primary TfdB-harboring phylum, whereas only a few homologs were derived from *Actinomycetota* and *Acidobacteriota* (Fig. 5b). Furthermore, TcsC homologs were widespread in TfdB-carrying strains, suggesting a broad microbial capacity for degrading brominated catechols. Based on the taxonomic distribution of putative BDE-47 degradation enzymes, it can be hypothesized that *Pseudomonadota* represents the primary bacterial taxa responsible for BDE-47 degradation in environments.

The environmental distribution of putative BDE-47 degradation enzymes was determined based on sample information manually retrieved from NCBI database. These candidate enzymes were distributed throughout a wide range of ecosystems, from natural environmental habitats (terrestrial, marine, and freshwater) to host-associated niches and engineered systems (Fig. 5c). Two TcsAaAb homologs were identified from wastewater, with no wastewater-originating TfdB homologs detected. Consistent with the soil origin of the TfdB-harboring strain JMP134, we found that soil is a dominant source for TfdB homologs. The co-occurrence of TcsAaAb and TfdB homolog was found in diverse environments, including soil, seawater, marine sediment, marine plastic particle, and marine host-associated microbiomes (such as coral reef, fish, and sponge). Combining the pervasive presence of downstream catabolic enzyme TcsC in bacteria containing TcsAaAb or TfdB, it is reasonable to postulate that microbial communities in these environments possess a synergistic capacity for BDE-47 degradation.

**Figure 6:**
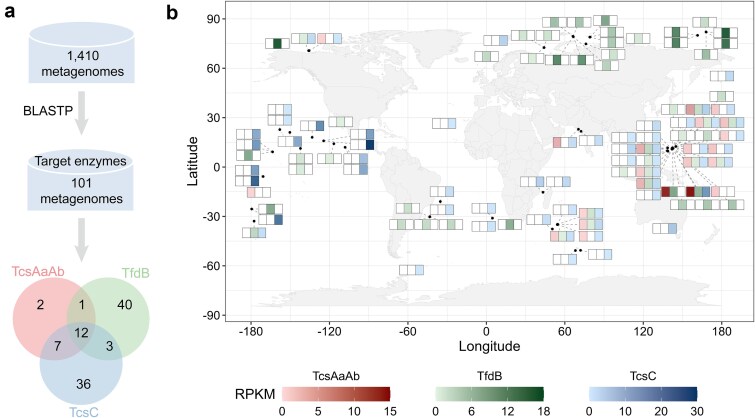
Global distribution patterns of the key functional genes involved in BDE-47 degradation across marine metagenomes. (a) Identification and co-occurrence analysis of genes involved in BDE-47 degradation. The numbers in the Venn diagram represent the number of metagenomic samples containing the target genes. (b) Biogeographical distribution and relative abundance of genes encoding target enzymes (TcsAaAb, TfdB, and TcsC) for BDE-47 degradation in the global ocean. The map was generated using the maps package in R. Each triple-square block represents a marine metagenomic sample.

Although analysis against the NR database confirms the presence of these key functional genes across diverse marine niches, their biogeographic distribution and relative abundance across the global ocean remain to be investigated. Hence, we interrogated 1410 marine metagenomes to resolve the global distribution patterns of genes encoding TcsAaAb, TfdB, and TcsC (Table S9). Results indicated that the presence of the functional genes involved in BDE-47 degradation in 101 marine metagenomes, and the genes encoding initial dioxygenase TcsAaAb, exhibited a much more limited distribution compared to those encoding hydroxylase TfdB and the halogenated catechol dioxygenase TcsC (Fig. 6a). The peak abundance of the TcsAaAb-encoding gene (RPKM = 13.61) was detected in a metagenomic sample from the Mariana Trench (MT) (Fig. 6b, Table S9). Moreover, the co-occurrence of TcsAaAb, TfdB, and TcsC genes was restricted to only 12 marine samples, 9 of which were sourced from the MT, spanning an extraordinary vertical gradient from the surface (0 m) to the hadal depths (10 905 m). As the known deepest site of the global ocean, the MT serves as a terminal sink of marine pollutants, with several studies documenting the accumulation of anthropogenic PBDEs within its hadal sediments [70, 71]. Despite its extreme depth, evident benthic oxygen consumption was observed at Challenger Deep in the MT, indicating active aerobic microbial metabolism in hadal sediments [72]. Furthermore, microbial communities in the MT exhibit robust metabolic activities toward alkanes and halogenated organic compounds [73, 74]. These signatures collectively support the plausibility of aerobic PBDE metabolism in this hadal environment. These findings corroborate the occurrence of enzymes involved in BDE-47 degradation across diverse marine provinces, revealing the MT may serve as a reservoir of microorganisms with PBDE degradation potential.

### Implications for environmental bioremediation and biogeochemical cycling

The mechanisms underlying the aerobic catabolism of PBDEs remain poorly understood, hindering the development of effective bioremediation strategies and the assessment of PBDEs environmental fate. This study advances current understanding through three key findings: (i) the complete degradation of BDE-47 can be achieved through metabolic relay within a synthetic consortium comprising two environmental isolates, which exhibited robust performance in authentic wastewater samples, including under an environmentally relevant concentration (Fig. S16). (ii) The molecular basis of this synergistic catabolism was validated via heterologous expression and biochemical characterization, providing a suite of biomarkers to assess the potential of BDE-47 degradation in the environment. (iii) Bioinformatic analyses revealed the widespread co-occurrence of these catabolic enzymes across diverse natural habitats, with their prevalence being significantly higher in the MT than in other regions of the global ocean.

So far, a fair number of studies on conceptual bioremediation have relied on genetically engineered strains [19], which face substantial barriers to *in situ* application due to concerns regarding ecological safety, regulatory restrictions, and limited environmental adaptability [75, 76]. In contrast, the synthetic consortium employed for BDE-47 degradation in this study was composed of environmentally isolated, naturally occurring degraders, ensuring high ecological adaptability and avoiding biosafety concerns. Unlike the extensively studied anaerobic debromination pathways mediated by reductive dehalogenases [20, 74], the genetic determinants driving aerobic PBDE catabolism remain largely enigmatic. Here, we functionally characterized TcsAaAb as an aerobic enzyme responsible for initiating BDE-47 degradation, providing mechanistic insight into the genetic basis of aerobic PBDE catabolism. Future investigations could evaluate its catalytic potential toward a broader range of PBDE congeners or employ protein engineering to expand its substrate range for the efficient elimination of diverse PBDEs [77].

Ecological analysis indicates that the assembled catabolic process is not an isolated phenomenon; rather, these catabolic elements involved in the synergistic degradation are distributed across diverse environments. This suggests that a substantially greater diversity of strains with BDE-47 degradation capacity and associated catabolic genes awaits exploration in both terrestrial and marine ecosystems. Furthermore, certain single microorganisms may possess the capacity to independently degrade BDE-47 through pathways analogous to that utilized by the synthetic consortium. Global marine metagenomic analysis revealed that microorganisms in the MT have the potential to catabolize PBDEs through the TcsAaAb-initiated aerobic pathway, highlighting the crucial yet overlooked role of the hadal biosphere in driving the global biogeochemical cycling of anthropogenic PBDEs.

## Supplementary Material

Supplementary_material_wrag163

## Data Availability

The complete genome assembly of *Rhizorhabdus wittichii* YL-JM2C is available in the NCBI database under BioProject PRJNA1397584. All raw data of amplicon sequencing has been deposited in the NCBI database under the BioProject PRJNA1413899.
