# Supplementary material for: Metabolic interactions enable aerobic degradation of the environmental pollutant BDE-47
Source: ISME J. 2026 Jun 27;20(1):wrag163. doi: 10.1093/ismejo/wrag163 (PMC13440307; doi:10.1093/ismejo/wrag163)
Supplement: Supplementary file 1 [file supplementary_material_wrag163.zip › Supporting_Information_wrag163.docx]

## Supporting Information

**Metabolic interactions enable aerobic degradation of the environmental pollutant BDE-47**

Running title: Synergistic aerobic degradation of BDE-47

Piaopiao Pan, Ning-Yi Zhou^*^

State Key Laboratory of Microbial Metabolism, Joint International Research Laboratory of Metabolic and Developmental Sciences, and School of Life Sciences and Biotechnology, Shanghai Jiao Tong University, 200240 Shanghai, China

^*^Correspondence to Ning-Yi Zhou, Email: ningyi.zhou@sjtu.edu.cn

**Number of Pages: 24**

**Number of Figures: 16**

**Number of Tables: 9 (Tables 6-9 in the file titled "Supplemental Tables 6-9")**

**Supplemental Figures:**

**Figure S1.** LC-MS analysis of BDE-47 transformation intermediate by strain YL-JM2C. The left panel shows the extracted ion chromatogram peaks of the 2,4-DBP standard and the product captured during whole-cell biotransformation, while the right panel presents their corresponding mass spectra.

**Figure S2.** Whole-cell biotransformation of BDE-47 by triclosan-induced and uninduced (glucose-pretreated) cells of strain YL-JM2C. The residual BDE-47 concentration was determined at different time points using HPLC. Data are presented as mean ± standard deviation (n = 3).

**Figure S3.** Transformation of BDE-47 by *E*. *coli* cells expressing dioxygenase TcsAaAb. (A) Proposed mechanism for the TcsAaAb-mediated transformation of BDE-47. The compound in the bracket was not detected. (B-C) Identification of BDE-47 transformation products through GC-MS. Extracted ion chromatogram peaks and mass spectra for 3,5-DBC (B) and 2,4-DBP (C) are shown.

**Figure S4.** Functional characterization of purified TcsC. (A) Reaction scheme of 3,5-DBC biotransformation by TcsC. (B) SDS-PAGE analysis of TcsC expression and purification. Lane M, protein marker; lane 1, cell extract of *E. coli* BL21(DE3) harboring pET-*tcsC*; lane 2, protein flow-through fluid; lanes 3-6, elution buffer containing 250 mM imidazole. The arrow indicates purified TcsC. (C) Spectra of reaction mixtures containing Tris-HCl buffer and 3,5-DBC (negative control). (D) Spectra of reaction mixtures containing Tris-HCl buffer, 3,5-DBC and purified TcsC. (E) Extracted ion chromatogram peak corresponding to the ring-cleavage product generated by TcsC. (F) Mass spectrum of the ring-cleavage product.

**Figure S5.** Functional characterization of the downstream enzymes TcsD, TcsE, and TcsF involved in the conversion of 2,4-dibromomuconate to 3-oxoadipate. Reactions were performed using crude cell extracts prepared from *E*. *coli* expressing the respective enzymes. Schematic representation of the reactions catalyzed by TcsD, TcsE, and TcsF is shown at the top. (A-C) UV-visible spectral changes during the conversion of 2,4-dibromomuconate to 2-bromodienelactone (A), 2-bromodienelactone to 2-bromomaleylacetate (B), and 2-bromomaleylacetate to 3-oxoadipate (C). (D-F) Extracted ion chromatogram peaks of the reaction products. (G-I) Corresponding mass spectra confirming the identities of 2-bromodienelactone, 2-bromomaleylacetate, and 3-oxoadipate.

**Figure S6.** Functional characterization of PcaIJ and PcaF from *R*. *wittichii* YL-JM2C involved in the 3-oxoadipate pathway. (A) Proposed reactions catalyzed by PcaIJ and PcaF for the conversion of 3-oxoadipate into central carbon metabolism intermediates. (B) UV spectrum scanning of 3-oxoadipate transformation catalyzed by cell extract of recombinant *E*. *coli* expressing PcaIJ. The arrow indicates the formation of 3-oxoadipyl-CoA-Mg^2+^ complex (305 nm). (C) UV spectrum scanning of 3-oxoadipyl-CoA cleavage catalyzed by cell extract of recombinant *E. coli* expressing PcaF. The arrow indicates the disappearance of 3-oxoadipyl-CoA-Mg^2+^ complex.

**Figure S7.** The *tfd* gene cluster and 2,4-D degradation pathway in *Cupriavidus necator* JMP134.[1] (A) Organization of the *tfd* genes on plasmid pJP4. Arrows indicate the sizes and orientations of all *tfd* genes currently known. (B) Overview of 2,4-D degradation pathway. The blue-colored intermediate is a structural analog of 2,4-DBP.

**Figure S8.** HPLC chromatograms of 2,4-DBP and 3,5-DBC standards, alongside the biotransformation products of 2,4-DBP by recombinant *E*. *coli* BL21(DE3) cells expressing TfdBI (BL21-TfdBI), TfdBII (BL21-TfdBII), or RwTfdB (BL21-RwTfdB), respectively.

**Figure S9.** SDS-PAGE analysis of purified TfdB homologs. Lane M1 and M2, protein marker; lane 1, crude extract supernatant of *E. coli* BL21(DE3) pET-*rwtfdB*; lane 2, pellet from the *E. coli* BL21(DE3) pET-*rwtfdB* lysate; lanes 3, flow-through from the *E. coli* BL21(DE3) pET-*rwtfdB* lysate; lane 4-5, RwTfdB eluted with 250 mM imidazole; lane 6, crude extract supernatant of *E. coli* BL21(DE3) pET-*tfdBI*; lane 7, TfdBI eluted with 250 mM imidazole; lane 8, crude extract supernatant of *E. coli* BL21(DE3) pET-*tfdBII*; lane 9, pellet from the *E. coli* BL21(DE3) pET-*tfdBII* lysate; lanes 10, flow-through from the *E. coli* BL21(DE3) pET-*tfdBII* lysate; lane 11, TfdBII eluted with 250 mM imidazole.

**Figure S10.** Growth curve of strain JMP134 on 2,4-dibromophenol (2,4-DBP). Strain JMP134 was cultivated in MSM supplemented with 100 μM 2,4-DBP as the sole carbon source, and growth was monitored by measuring OD_600_ over time. A substrate-free culture served as the negative control.

**Figure S11.** Comparison of BDE-47 degradation efficiency between the transwell and mixed groups over time. Two-tailed Student's t tests were performed for statistical analysis. ns indicates no significant difference (*P* ≥ 0.05) between the two groups at the corresponding time point. No accumulation of 2,4-DBP was detected in either group throughout the incubation process. The transwell assays were performed in a 24-well transwell plate (0.4 μm pore size; Jet Biofil). For transwell group, 100 μL of strain JMP134 suspension (OD₆₀₀ = 0.25) was added to the upper insert, and the lower chamber with 100 μL of strain YL-JM2C suspension (OD₆₀₀ = 0.25) plus 300 μL of MSM, supplemented with BDE-47 to a final concentration of 20 µM. For the Mixed group (without physical separation), the same amounts of JMP134 and YL-JM2C suspensions (100 μL each, OD₆₀₀ = 0.25) were co-inoculated directly into a single well of a 24-well plate containing 300 μL of MSM supplemented with 20 µM BDE-47.

**Figure S12.** Dynamics of the two bacterial strains during BDE-47 degradation. The BDE-47 concentration is shown as line, and bacterial abundance is shown as histogram by cell densities (cells/mL). Blue bars represent strain YL-JM2C, and pink bars represent strain JMP134. Both strains were inoculated at 5% (v/v) from culture with an OD_600_ of 1.0 into MSM supplemented with 50 μM BDE-47, and replenished with another 50 μM BDE-47 on day 5 and 10. Bacterial abundances were determined by qPCR targeting a strain-specific region of the 16S rRNA gene after normalization against the 16S rRNA gene copy numbers per genome (six copies for strain JMP134 and two copies for strain YL-JM2C).

**Figure S13.** Effects of temperature (A) and pH (B) on BDE-47 degradation by the synthetic consortium. The BDE-47 degradation percentage was determined after a 12-h incubation. The initial concentration of BDE-47 was 50 μM, with the bacterial culture inoculated at an OD_600_ of 4.0.

**Figure S14.** Conserved domain analysis and phylogenetic analysis of TcsAa homologs retrieved from NR database at 35% identity threshold. (A) Multiple sequence alignment of TcsAa and retrieved homologs. Residues labeled with position numbers are involved in Rieske-type [2Fe–2S] cluster binding and mononuclear Fe²⁺ coordination. Residue positions are numbered according to the TcsAa sequence. (B) Maximum-likelihood phylogenetic analysis of TcsAa homologs and functionally characterized Rieske dioxygenases.

**Figure S15.** Phylogenetic tree of TfdB homologous proteins. The sequences were retrieved via BLASTP against NR database with an identity threshold of 50%. Proteins TfdBI and TfdBII are marked with green circles, and RwTfdB is marked with a red circle. The branch highlighted in orange represents the clade containing TfdBI and TfdBII, and the protein sequences within this clade are proposed to be potential candidates possessing activity towards 2,4-DBP.

**Figure S16.** BDE-47 degradation and bromide ion release by the synthetic consortium. Both strains were adjusted to an OD_600_ of 1.0 and inoculated at 5% (v/v, each). Control experiments were prepared with autoclaved cell suspensions under same conditions. (A) BDE-47 degradation by the synthetic consortium in MSM supplemented with 0.4 μM BDE-47. Residual BDE-47 concentrations were quantified by GC-MS. The degradation of BDE-47 by the synthetic consortium followed a first-order kinetic model (ln(C/C₀) = -kt), with a rate constant (k) of 0.44 h^-1^. (B) Bromide ion release corresponding to the degradation experiment shown in (A). Bromide ion concentrations were quantified by ion chromatography at 0 h and 12 h. (C) BDE-47 degradation by the synthetic consortium in activated sludge microcosms. The microcosms were established using activated sludge collected from WW1 as previously described [2]. The final sludge concentration of the microcosms was 1,580 mg/L, and BDE-47 was amended at 0.4 μM, corresponding to 123 mg BDE-47/kg dry solids, which falls within the range of concentrations previously reported in contaminated environments [3].

**Supplemental Tables:**

**Table S1.** Bacterial strains and plasmids used in this study.

| **Strains or plasmid** | **Description and characteristics** | **Source** |
| --- | --- | --- |
| Strains |  |  |
| *Rhizorhabdus wittichii* YL-JM2C | Triclosan degrader | [4] |
| *Cupriavidus necator* JMP134 | 2,4-dichlorophenoxyacetic acid degrader | [5] |
| *E. coli* DH5α | *supE44 lacU169(ϕ80lacZΔM15) hsdR17 recA1 endA1 gyrA96 Δthi relA1* | [6] |
| *E. coli* BL21(DE3) | *F^–^ ompT hsdS_B_ (r_B_ – m_B_ –) gal dcm lacY1 (DE3)* | [7] |
| Plasmids |  |  |
| pET-28a(+) | IPTG inducible expression vector; Km^r^ | Novagen |
| pET-*tcsAaAb* | *tcsAaAb* fragment inserted into pET-28a(+) between NdeI and BamHI; Km^r^ | This study |
| pET-*tcsC* | *tcsC* fragment inserted into pET-28a(+) between NdeI and BamHI; Km^r^ | This study |
| pET-*tfdBI* | *tfdBI* fragment inserted into pET-28a(+) between NdeI and BamHI; Km^r^ | This study |
| pET-*tfdBII* | *tfdBII* fragment inserted into pET-28a(+) between NdeI and BamHI; Km^r^ | This study |
| pET-*rwtfdB* | *rwtfdB* fragment inserted into pET-28a(+) between NdeI and BamHI; Km^r^ | This study |

**Table S2.** Primers used in this study.

| **Primer** | **Sequence (5’→3’)** | **Purpose** |
| --- | --- | --- |
| pET-*tcsAaAb*-F | TGCCGCGCGGCAGCCATATGATGGCGACGCGGG | Amplification of *tcsAaAb* gene for expression |
| pET-*tcsAaAb*-R | GGAGCTCGAATTCGGATCCTTAGACGTCGAGCACGAGCT |  |
| pET-*tcsC*-F | TGCCGCGCGGCAGCCATATGATGTCGGATGCTGAACGACTGGCA | Amplification of *tcsC* gene for expression |
| pET-*tcsC*-R | GGAGCTCGAATTCGGATCCTCAGGCTGCCTCGG |  |
| pET-*tfdBI*-F | CGCGCGGCAGCCATATGATGGCATTGACGATCGAAACCGA | Amplification of *tfdBI* gene for expression |
| pET-*tfdBI*-R | GCTCGAATTCGGATCCTTATATAGCTCGAGACGTAACGCG |  |
| pET-*tfdBII*-F | CGCGCGGCAGCCATATGATGAACGAAAAAGCCAACACCGTC | Amplification of *tfdBII* gene for expression |
| pET-*tfdBII*-R | GCTCGAATTCGGATCCTCAGTGCGCGCAGAGCGC |  |
| pET-*rwtfdB*-F | CGCGCGGCAGCCATATGATGGCGAAAATCGTTGAAACCGA | Amplification of *rwtfdB* gene for expression |
| pET-*rwtfdB*-R | GCTCGAATTCGGATCCTCAGCCGGTCTTCGCGAT |  |
| pET-*tcsD*-F | CATCATCACAGCAGCGGCATGCTTCCCGTGACCGACCTCGCCAC | Amplification of *tcsD* gene for expression |
| pET-*tcsD*-R | TCGACGGAGCTCGAATTCCTAGCTCGGGCGCGTGAA |  |
| pET-*tcsE*-F | CATCATCACAGCAGCGGCGTGAACGACGCGATGCGG | Amplification of *tcsE* gene for expression |
| pET-*tcsE*-R | TCGACGGAGCTCGAATTCTCAAGGCTTAGCGATCGCTGCGT |  |
| pET-*tcsF*-F | CATCATCACAGCAGCGGCATGGACAGCTTCGTTTATGAGGGC | Amplification of *tcsF* gene for expression |
| pET-*tcsF*-R | TCGACGGAGCTCGAATTCTCAGCCCGCGTAGGT |  |
| pET-*pcaIJ-*JM2C-F | CATCATCACAGCAGCGGCATGTCATTGCCCGAGGCC | Amplification of *pcaIJ* gene for expression |
| pET-*pcaIJ-*JM2C-R | TCGACGGAGCTCGAATTCTCATGCGGCTCCCTGGT |  |
| pET-*pcaF-*JM2C-F | CATCATCACAGCAGCGGCGTGGCGGCGGCCTTTCT | Amplification of *pcaF* gene for expression |
| pET-*pcaF-*JM2C-R | TCGACGGAGCTCGAATTCTCACATACGTCCAAGCGCCA |  |
| pET-*tfdCIDIEIFI*-F | CATCATCACAGCAGCGGCGTGAACAAAAGAGTCAAGGATGTTGTCGATGC | Amplification of *tfdCIDIEIFI* gene for expression |
| pET-*tfdCIDIEIFI*-F | TCGACGGAGCTCGAATTCTTATTTGAAATCCGGTCTTCGCCCACGAAA |  |
| qRw16S-F | TCACTGGACGACTGTTGACG | Quantification of strain YL-JM2C |
| qRw16S-R | TTTAACCTTGCGGCCGTACT |  |
| qCn16S-F | GGCAGCAGTGGGGAATTTTG | Quantification of strain JMP134 |
| qCn16S-R | CACGTAGTTAGCCGGTGCTT |  |

**Table S3.** Details of WWTP samples from different cities across China.

| **Sample name** | **Source (treatment capacity)** | **Location** | **Latitude and longitude** |
| --- | --- | --- | --- |
| Wastewater 1 (WW1) | Municipal WWTP (240,000 m^3^ per day) | Lanzhou city, Gansu Province | 103.71^o^E, 36.09^o^N |
| Wastewater 2 (WW2) | Municipal WWTP (150,000 m^3^ per day) | Jiading Distinct, Shanghai city | 121.38^o^E, 31.11^o^N |
| Wastewater 3 (WW3) | Municipal WWTP (100,000 m^3^ per day) | Wuhan city, Hubei Province | 114.43^o^E, 30.49^o^N |

**Table S4.** Pairwise identity of TfdBI, TfdBII, and RwTfdB.

| **Query** | **Subject** | **Identity** |
| --- | --- | --- |
| RwTfdB (WP_029992645.1) | TfdBI  (WP_011178382.1) | 52.53% |
| RwTfdB  (WP_029992645.1) | TfdBII (WP_011178390.1) | 55.60% |
| TfdBI  (WP_011178382.1) | TfdBII (WP_011178390.1) | 63.52% |

**Table S5.** Chromatographic conditions for the detection of compounds used in this study.

| **Compound** | **Mobile phase** | **Elution program** | **Flow rate**  **(mL/min)** | **Column** |
| --- | --- | --- | --- | --- |
| BDE-47, 2,4-DBP, 3,5-DBC | 0.1% formic acid in water (A) and acetonitrile (B) | 0-7 min, 30%-100% B; 7-16 min, 100% B; 16-18 min, 100-30% B; 18-20 min, 30% B | 0.8 (HPLC) | C18 |
| BDE-47, 2,4-DBP, 3,5-DBC | 0.1% formic acid in water (A) and acetonitrile (B) | 0-10 min, 50%-100% B; 10-25 min, 100% B; 25-28 min, 100%-50% B; 28-30 min, 50% B. | 0.4 (LC-MS) | C18 |
| 2,4-Dibromomuconate, 2-Bromodienelactone, 2-Bromomaleylacetate | 10 mM ammonium formate (A) and acetonitrile (B) | 0-3 min, 5% B; 3-9 min, 5%-45% B; 9-12 min, 45%-80% B; 12-14 min, 80% B; 14-16 min, 80%-5% B | 0.3 (LC-MS) | C18 |
| 3-Oxoadipate | 10 mM ammonium formate (A) and acetonitrile (B) | 0-5 min, 90%-80% B; 5-10 min, 80%-60% B; 10-15min, 60%-20% B; 15-18 min, 20%-90% B | 0.3 (LC-MS) | HILIC |

**References**

1.	Laemmli CM, Leveau JHJ, Zehnder AJB, et al. Characterization of a second *tfd* gene cluster for chlorophenol and chlorocatechol metabolism on plasmid pJP4 in *Ralstonia eutropha* JMP134(pJP4). *J Bacteriol* 2000;**182**:4165–4172. https://doi.org/10.1128/jb.182.15.4165-4172.2000

2.	Xu G, Zhao S, He J. Underexplored organohalide-respiring bacteria in sewage sludge debrominating polybrominated diphenyl ethers. *Environ Sci Technol* 2024;**58**:13348–13357. https://doi.org/10.1021/acs.est.4c00906

3.	Huang C, Zeng Y, Luo X, et al. In situ microbial degradation of PBDEs in sediments from an E-waste site as revealed by positive matrix factorization and compound-specific stable carbon isotope analysis. *Environ Sci Technol* 2019;**53**:1928–1936. https://doi.org/10.1021/acs.est.8b06110

4.	Mulla SI, Wang H, Sun Q, et al. Characterization of triclosan metabolism in *Sphingomonas* sp. strain YL-JM2C. *Sci Rep* 2016;**6**:21965. https://doi.org/10.1038/srep21965

5.	Lykidis A, Pérez-Pantoja D, Ledger T, et al. The complete multipartite genome sequence of *Cupriavidus necator* JMP134, a versatile pollutant degrader. *PLOS One* 2010;**5**:e9729. https://doi.org/10.1371/journal.pone.0009729

6.	Hanahan D. Studies on transformation of *Escherichia coli* with plasmids. *J Mol Biol* 1983;**166**:557–580. https://doi.org/10.1016/s0022-2836(83)80284-8

7.	Studier FW, Moffatt BA. Use of bacteriophage T7 RNA polymerase to direct selective high-level expression of cloned genes. *J Mol Biol* 1986;**189**:113–130. https://doi.org/10.1016/0022-2836(86)90385-2
